# Impact of Annealing Treatment on the Microstructure and Micromechanical Properties of Pb-Containing and Pb-Free Solder Alloys

**DOI:** 10.3390/ma18112596

**Published:** 2025-06-02

**Authors:** Wen Jiang, Changwei Wang, Kangning Han, Yaxin Zhu, Chuantao Hou, Ruisi Xing

**Affiliations:** 1 Department of Engineering Mechanics, School of Aerospace Engineering, Huazhong University of Science and Technology, Wuhan 430074, China; d202080498@hust.edu.cn (W.J.); wangchangwei@hust.edu.cn (C.W.); 15930773616@163.com (K.H.); 2 Hubei Key Laboratory of Engineering Structural Analysis and Safety Assessment, Wuhan 430074, China; 3 Science and Technology on Reliability and Environmental Engineering Laboratory, Beijing Institute of Structure and Environment Engineering, No 1 South Dahongmen Road, Beijing 100076, China; houcht702@163.com (C.H.); xingruisi1108@163.com (R.X.)

**Keywords:** Sn-Ag-Cu, Sn-Pb, annealing, microstructure, nanoindentation

## Abstract

This study investigates the microstructural changes and micromechanical responses of Pb-containing and Pb-free solder alloys subjected to various annealing conditions, with the goal of elucidating the relationship between microstructure evolution and micromechanical properties. Results indicate that grain size in SAC0307 and SAC305 significantly increases with annealing temperature, while that of Sn63Pb37 remains relatively stable. In Sn63Pb37, the Pb-rich phase coarsens and its area fraction increases with higher annealing temperatures, whereas in SAC0307, the intermetallic compounds (IMCs) phase coarsens but its area fraction decreases. Nano-indentation tests show that the hardness of Sn63Pb37 significantly increases with rising annealing temperature, whereas the hardness of SAC0307 decreases, and that of SAC305 remains relatively unchanged. These variations in these alloys induced by annealing are closely related to the changes in the hardness of individual phases within the grains. For Sn63Pb37, higher annealing temperatures increase the hardness of both the Sn matrix and Pb-rich phases, enhancing overall hardness. Conversely, in SAC0307, increased temperatures reduced the hardness of both the Sn matrix and IMCs phases, resulting in lower overall hardness. The differing trends in mechanical property of individual phases in three alloy are attributed to their distinct evolutions under annealing treatment. This study provides insights into the micromechanical behavior of solder alloys under annealing and offers guidance for optimizing their performance.

## 1. Introduction

Microelectronic packaging technology forms the foundation of the electronics industry [1,2,3,4]. Since the advent of integrated circuits, microelectronics technology has evolved significantly over the past 70 years. To meet the demands for miniaturization, high speed, and high reliability in electronic systems, the integration of electronic components has become essential, thus increasing the importance of electronic packaging technology. In electronic packaging systems, solder joints play critical roles by providing mechanical support, electrical connections, and thermal conduction between electronic components and printed circuit boards. Therefore, the reliability of packaging materials, particularly the solders used, is crucial for the proper functioning of microelectronic devices and equipment [5,6]. Solders are primarily categorized into lead-containing (Pb-containing) and lead-free (Pb-free) types. Pb-containing solder, typically referring to the Sn-Pb alloy, exhibits advantageous properties such as a low melting point, good fluidity, ease of handling, cost-effectiveness, and high reliability [7]. As a result, Sn-Pb solder is widely employed in various electronic manufacturing industries. However, due to the inherent toxicity of Pb and its harmful effects on the environment and human health, there has been a global initiative to minimize or eliminate the use of Pb in solder [8,9]. Consequently, the transition towards Pb-free electronic packaging has become essential, leading to substantial efforts in the development of Pb-free solder alloys [10,11,12,13,14,15,16]. Nevertheless, Pb-containing solder is still irreplaceable in certain special fields (military and aerospace industries) at present. The main reason is that under the high reliability requirements of military and aerospace equipment (such as long-term service and extreme environments), the performance of traditional Pb-containing solder is more stable [17,18,19]. Among various Pb-free alloy systems, the Sn-Ag-Cu alloy has emerged as one of the most promising candidates due to its relatively low melting temperature, commendable mechanical properties, and excellent compatibility with other components [20,21]. As representatives of Sn-Ag-Cu alloys, both SAC305 and SAC0307 are commonly used Pb-free solders at present. SAC305 has a high Ag content, excellent mechanical properties, but a relatively high cost. SAC0307, on the other hand, has a low Ag content and slightly inferior mechanical properties, and yet has a lower cost [22].

Both Pb-containing and Pb-free solders display multi-scale microstructures ranging from the nanoscale to the macroscale. Within the grains, Sn phases exhibit dendritic features, including primary and secondary dendrites, with sizes ranging from tens to hundreds of micrometers. Between the secondary dendrites, Sn and alloying elements form nanoscale eutectic structures (e.g., Ag_3_Sn, Zn_9_Sn, Bi_58_Sn, and so on) [23,24]. Variations in these microstructures can significantly impact the macroscopic properties of the solder, including strength, hardness, creep, fatigue, and other performance characteristics [21,25,26,27,28,29,30,31,32]. For instance, adding a small amount of Zn to Sn-0.7Cu solder refines its microstructure, resulting in smaller grain sizes [33]. The inclusion of Zn also reduces the driving force for the formation of intermetallic compounds (IMCs), enhancing structural stability and mechanical properties. In Sn-Ag-Cu (SAC) solder, the presence of Cu_3_Sn during service often promotes the formation of microvoids, leading to brittle failure. Alloying elements such as Fe, Co, and Ni inhibit the growth of Cu_3_Sn, thus enhancing the solder joint’s resistance to failure [34,35]. These findings suggest possible methods for improving the mechanical properties and long-term reliability of solders [31,34,36].

Both annealing treatments and thermal aging are well-established factors that profoundly influence the microstructure evolution in solders and solder joints. During annealing or thermal aging processes, various microstructural changes occur, including particle coarsening, grain rotation, and sub-grain formation [32], all of which can significantly alter the properties of the solder alloy. For example, the functional properties of NiTi shape memory alloys are dependent on grain size [37]. Annealing procedures are typically employed to achieve a homogeneous microstructure and alleviate residual stresses. However, Long et al. observed a significant decrease in the yield stress of SAC305 solder with increasing annealing duration, particularly at higher temperatures [27,38]. This discovery prompts investigation into the underlying mechanisms responsible for this phenomenon. Potential factors contributing to the yield stress reduction include changes in particle distribution and size within the solder matrix, alterations in grain boundary characteristics, the influence of temperature on diffusion kinetics, among others. Further research is essential to fully elucidate these mechanisms. Thermal aging also exerts a substantial impact on the microstructure of both Pb-containing (96Sn-04Pb) and Pb-free (96.5Sn-3.0Ag-0.5Cu) solders [32]. A notable effect of thermal aging is the enlargement of the average size of IMCs, which contributes to microstructural coarsening. Among them, Fix et al. [39] have investigated the growth behavior of Cu_6_Sn_5_ particles in SAC solder during solid-state aging, and found that the growth mechanism is diffusion-controlled and that different dominant diffusion modes are exhibited at varying temperatures. IMCs are known for their hardness and brittleness, and their increased size renders solders more susceptible to failure, thereby reducing yield stress and ultimate tensile stress in both Pb-based and Pb-free solders. Furthermore, microstructural changes induced by thermal aging and annealing significantly influence the creep resistance and fatigue properties of solder and solder joints [25,26,40]. Consequently, a comprehensive understanding and control of solder microstructure are crucial for tailoring desired mechanical characteristics in solder alloys for specific applications.

A thorough understanding of the microstructural changes and their impact on the mechanical properties of solders is essential for designing and ensuring the reliability of solder joints in various applications. Numerous previous studies [17,41,42,43,44,45,46,47] have delved into the microstructural evolution and mechanical property alterations of diverse solder alloys, including SAC, SnPb, and Sn-Ag-Zn, under heat treatment (annealing) conditions. These investigations employed various methodologies, such as uniaxial tensile testing, metallographic analysis, electron microscopy observation, energy dispersive spectroscopy (EDS) analysis, and theoretical modeling. Notably, despite this extensive research, low-Ag-content SAC solder alloys, such as SAC0307, have received relatively little attention. The existing studies predominantly focus on macroscopic mechanical properties, leaving a significant research gap in comprehensively exploring the intricate relationship between the solder microstructure and micro-mechanical properties. This gap is particularly notable given the presence of abundant Pb-rich phases, Sn phases, and eutectic phases at the microscale. The mechanical properties of both Pb-containing and Pb-free solders are influenced by the distinct properties of each individual phase as well as their synergistic effects [36,40]. Therefore, investigating the micromechanical properties of solders is crucial. Unfortunately, research focusing on these micro-mechanical properties, particularly the properties of each individual phase in solder alloys, remains inadequate. In this study, we fill this gap by conducting the first comprehensive investigation of the microstructure and mechanical properties of individual phases in Pb-containing (Sn63Pb37) and Pb-free (SAC0307, SAC305) solder alloys under various annealing conditions. Nano-indentation testing [48], a widely used technique for measuring the mechanical properties of materials at small scales, provides a suitable method for studying the microscopic mechanical properties of solders. Motivated by these scientific and engineering requirements, we conducted various annealing processes on both Pb-containing (Sn63Pb37) and Pb-free (SAC0307 and SAC305) solder alloys, followed by nano-indentation tests. By examining the microstructural evolution resulting from the annealing processes, we elucidated the relationships between the microstructures and the micromechanical responses of Sn63Pb37, SAC0307, and SAC305 solders. The results enhance our understanding of the behavior and characteristics of solders, providing valuable guidance for optimizing the performance of both Pb-containing and Pb-free solder alloys.

## 2. Experiment Procedures

The objective of this study is to investigate how different microstructures affect the mechanical properties of both Pb-containing and Pb-free solder alloys. Specifically, three as-cast solder alloy specimens with dimensions of approximately 10.0 mm × 10.0 mm × 2.0 mm were subjected to annealing treatment: Sn63Pb37 (63% Sn and 37% Pb), SAC0307 (99% Sn, 0.3% Ag, and 0.7% Cu), and SAC305 (96.5% Sn, 3.0% Ag, and 0.5% Cu). The annealing process was carried out in a DHG-9030A drying oven, noted for its temperature stability of ±1.0 °C. Considering that the melting temperature *T_m_* of Sn63Pb37 solder is 183 °C, the annealing temperatures were 75 °C, 125 °C, and 165 °C. The durations were 6 h, 12 h, and 24 h. For ease of comparison, the Sn63Pb37, SAC0307, and SAC305 solders are all annealed at the same temperature and duration. This approach allows us to assess the impact of prolonged exposure to specific temperatures on the solder alloy, providing valuable insights into their characteristics and behavior under different annealing conditions. For simplicity, the annealing conditions are labeled as T(X)-D(Y), where X represents the annealing temperature and Y represents the duration. For example, an annealing treatment at 75 °C for 6 h is denoted as T75-D6.

After annealing treatments, the samples underwent slicing from the three annealed solder alloys for subsequent microstructure characterizations and nano-indentation tests. Initially, the samples were cold-embedded in epoxy resin and subjected to grinding and polishing. The initial polishing used SiC paper with grit sizes ranging from 400 to 1200 to achieve a smooth surface. Next, the sample surfaces underwent fine grinding with single crystal diamond suspension, reducing particle sizes gradually from 15 μm to 3 μm to eliminate any surface scratches effectively. Following this, the samples underwent polishing with oxidizing polymer solution (OPS) for approximately 30 min to induce a corrosion effect, removing the previous deformation layer and enhancing surface quality. Subsequently, the polished samples were cleaned using an ultrasonic cleaner and air-dried. To ensure microstructural stability, the samples were stored at room temperature for at least one day before microscopic analysis and testing. Additionally, a separate group of samples was prepared from the three as-cast solder alloys to enable a comparison between the annealed and original states of the solder alloys.

All prepared specimens were analyzed using the JSM-IT500 scanning electron microscope (SEM) equipped with energy dispersive spectroscopy (EDS) to examine the microstructural changes resulting from annealing treatments, enabling detailed observation at the microscopic level. Further characterization of the test specimens’ microstructures was conducted using a field emission scanning electron microscope (GeminiSEM300, Carl Zeiss, Oberkochen, Germany) equipped with an electron backscatter diffraction (EBSD) detector (Oxford Instruments Aztec Nordlys Max3, Bristol, UK). Nanoindentation testing was performed using the Hysitron TI 980 micro-mechanical test platform, employing displacement control mode with a maximum indentation depth set at 300 nm. Following established procedures [49,50], the indenter applied a constant speed for 5 s, held for 2 s, and then unloaded over 5 s. This loading protocol was consistently applied to both as-cast and annealed samples to ensure experimental consistency. Hardness and modulus were determined using indentation theory to analyze displacement–depth curves obtained from indentation measurements [51,52]. Given the material’s microstructural complexity, variations in hardness and modulus were expected due to local material inhomogeneities at the microscale. To account for these variations, dozens of indentations were performed on each sample to obtain statistically averaged hardness and modulus values. Combining the SEM observation, the indentations on individual phases within the solder can be distinguished. By correlating the indentation results with the identified phases, accurate quantification of nano-hardness and modulus for each specific phase in the solder alloys can be obtained.

## 3. Results and Discussion

### 3.1. Impacts of Annealing Treatments on the Microstructures

Investigating the impact of annealing treatments on solder alloys necessitates a thorough examination of internal microstructural changes under varying conditions. Through SEM analysis, we extensively studied how the annealing process alters microstructures, which is pivotal for comprehending its influence on solder alloy mechanical properties. Numerous studies have highlighted the significant effects of annealing treatments on grain morphology and size [46,53,54]. Therefore, our initial focus was on assessing how annealing affects the microstructure of SAC0307, SAC305, and Sn63Pb37 solders at the grain level.

**Figure 1:**
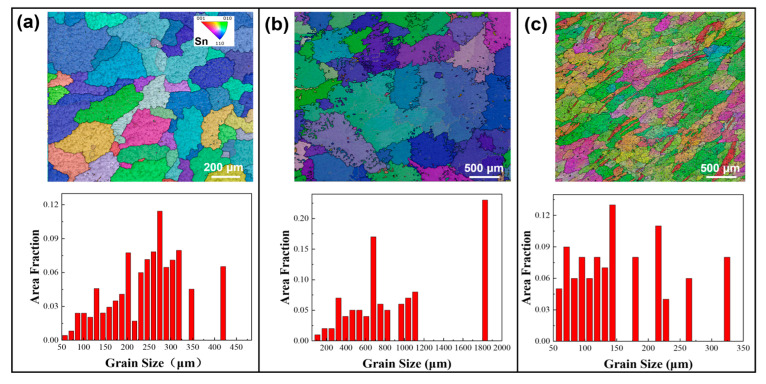
Grain morphologies and size statistics of as-cast (**a**) SAC0307, (**b**) SAC305, and (**c**) Sn63Pb37 solder alloys.

**Figure 2:**
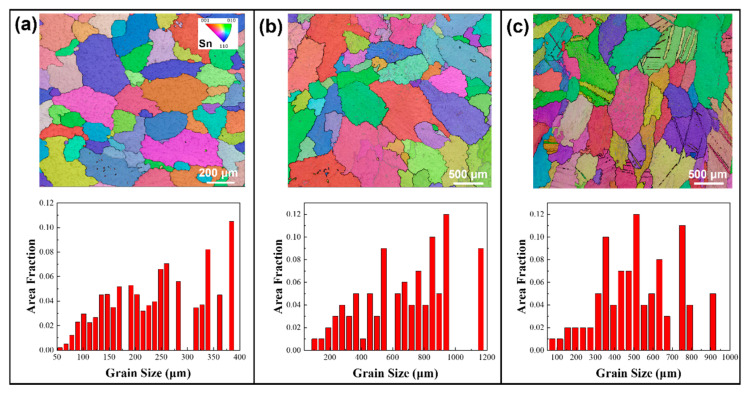
Grain morphologies and size statistics of SAC0307 solder after annealing at (**a**) 75 °C, (**b**) 125 °C, and (**c**) 165 °C.

**Figure 3:**
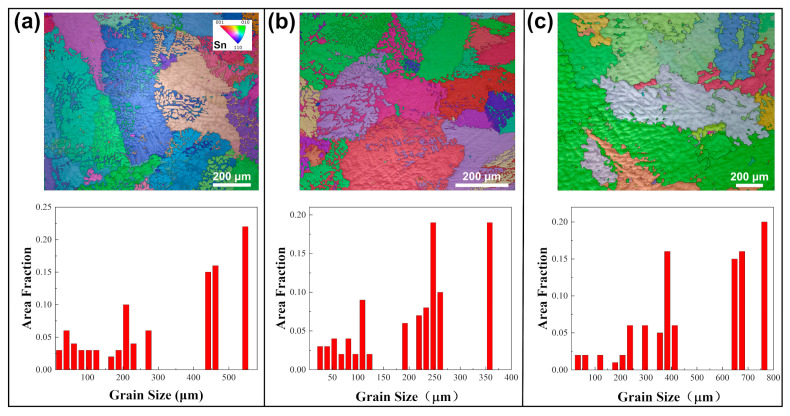
Grain morphologies and size statistics of SAC305 solder after annealing at (**a**) 75 °C, (**b**) 125 °C, and (**c**) 165 °C.

**Figure 4:**
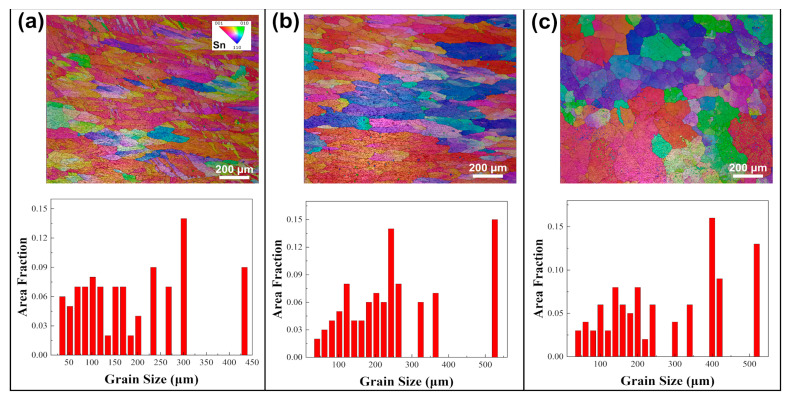
Grain morphologies and size statistics of Sn63Pb37 solder after annealing at (**a**) 75 °C, (**b**) 125 °C, and (**c**) 165 °C.

EBSD analyses were performed on alloy samples subjected to various annealing conditions. Figure 1 shows the inverse pole figure (IPF) maps and size distribution of different solders in their initial as-cast states. Grain sizes in this study are expressed in terms of equivalent circle diameter. Grain size measurements using EBSD adhere to ASTM E2627-13 (2013) and ISO 13067 (2011) [55,56], with this study following ISO 13067. Using the EBSD data processing software AztecCrystal 2.1, the average grain sizes in SAC0307, SAC305, and Sn63Pb37 solders were determined to be 246.8 μm, 921.8 μm, and 149.9 μm, respectively. Therefore, it can be concluded that under the initial as-cast conditions, SAC305 solder has the largest grain, with a maximum size exceeding 1800 μm (as shown in Figure 1b), followed by SAC0307, while Sn63Pb37 has the smallest average grain size, about one-sixth that of SAC305. Figure 2 illustrates the IPF maps and grain size distributions for the SAC0307 alloy after annealing at various temperatures. As shown in Figure 2a–c, the grains of the SAC0307 specimen coarsen noticeably with increasing annealing temperature. When the annealing temperature is raised from 75 °C to 165 °C, the average grain size increases from 240.7 μm to 533.8 μm. The grain size of SAC305 alloy exhibits a similar trend with increasing annealing temperature. As depicted in Figure 3, with the annealing temperature increasing from 75 °C to 165 °C, the average grain size of SAC305 alloy increased from 327.8 μm to 504.8 μm. Figure 4 presents the IPF maps and grain size distributions of Sn63Pb37 solder at various annealing temperatures. With the increase in annealing temperature, the dendritic grains in Sn63Pb37 solder gradually transform into nearly equiaxed grains. The grain size distributions in Figure 4a–c show that the average grain size in Sn63Pb37 solder increases with the increase in annealing temperature. The average grain sizes are 186.5 μm, 248.7 μm, and 275.1 μm, respectively. Recent research supports this phenomenon. During the annealing process, the material is in a high-temperature state, and the higher the temperature, the stronger the atomic diffusion ability, which promotes the movement of grain boundaries and makes the grains grow continuously [57]. Consequently, these differences in grain size response to annealing temperature necessarily leads to variations in the mechanical properties of the solder alloys, which will be further discussed in the subsequent section.

**Figure 5:**
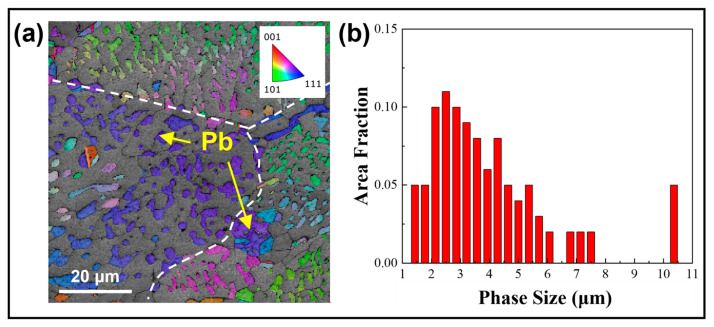
(**a**) Distribution and (**b**) size statistics of Pb-rich phases within the grains of Sn63Pb37.

**Figure 6:**
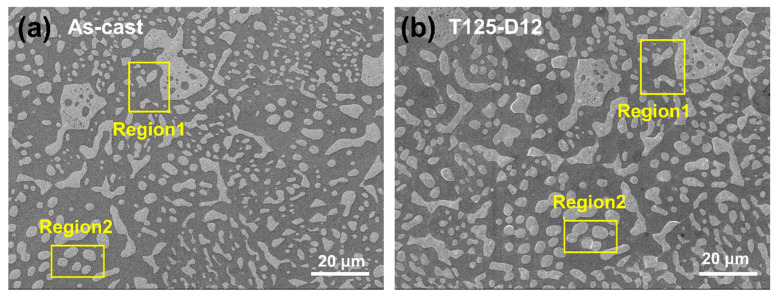
Coarsening and aggregation of Pb-rich phase in Sn63Pb37 at the same regions (**a**) before and (**b**) after annealing.

**Figure 7:**
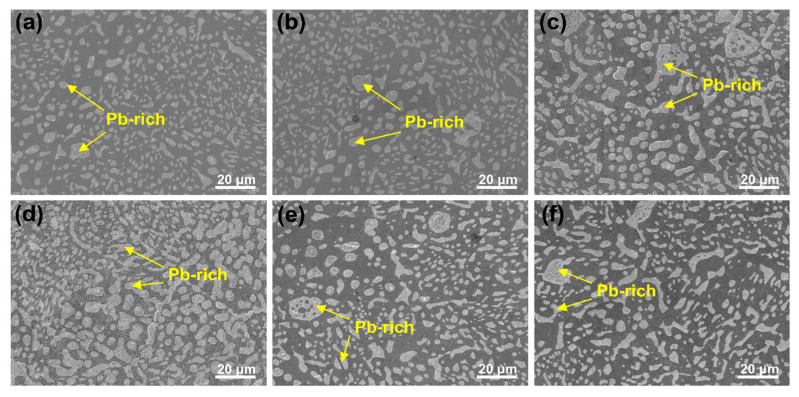
Morphologies of Pb-rich phases in Sn63Pb37 under various conditions: (**a**) as-cast, (**b**) T75-D12, (**c**) T125-D12, (**d**) T165-D12, (**e**) T125-D6, and (**f**) T125-24.

The annealing treatment significantly impacts not only the grain size but also the microstructure within the grain. As shown in Figure 5, Sn63Pb37 contains numerous separately distributed Pb-rich phases, ranging from about 2 to 10 μm in size, with an average size of 3.8 μm. The IPF in Figure 5a shows that the orientations of Pb-rich phases are similar within the same grain but differ across different grains. Under annealing, the Pb-rich phases exhibit coarsening and aggregation. For instance, Figure 6a,b display the microscopic morphologies of the same area in the as-cast sample and the sample annealed at 125 °C, respectively. In these images, the lighter regions correspond to the Pb-rich phase (eutectic phase), while the darker regions represent the Sn matrix phase. It is evident that the adjacent small-sized Pb-rich phases in regions 1 and 2, highlighted in the yellow boxes, have aggregated during annealing, resulting in the formation of larger Pb-rich phases. Figure 7 shows the morphologies of Pb-rich phases in Sn63Pb37 in its as-cast state and after different annealing treatments. Notably, irregularly shaped Pb-rich phases with diameters exceeding 10 μm and a dispersed distribution are observed in the as-cast sample. Additionally, black particles, likely Sn particles, are visible within these phases. Figure 7b–f illustrate the microscopic morphologies of the samples after various annealing treatments. As the annealing temperature increases (Figure 7b–d), the coarsening of Pb-rich phases becomes increasingly apparent. However, when the annealing temperature is fixed at 125 °C and the annealing duration is increased from 6 to 24 h (Figure 7c,e,f), the size of the Pb-rich phases shows minimal changes. This indicates that annealing temperature, rather than duration, predominantly controls the microstructure evolution in Sn63Pb37 alloy.

**Figure 8:**
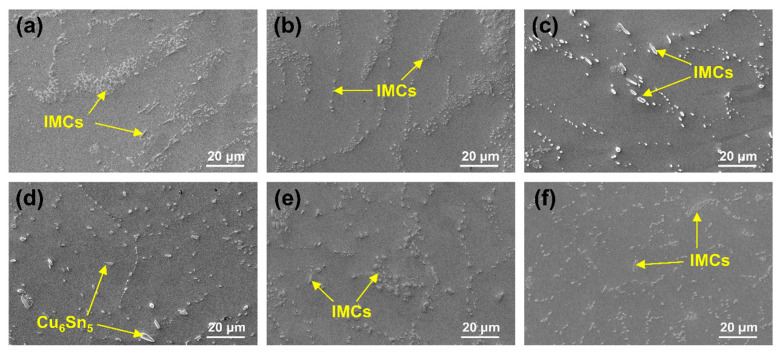
Morphologies within the grains of SAC0307 under various conditions: (**a**) as-cast, (**b**) T75-D12, (**c**) T125-D12, (**d**) T165-D12, (**e**) T125-D6, and (**f**) T125-24.

**Figure 9:**
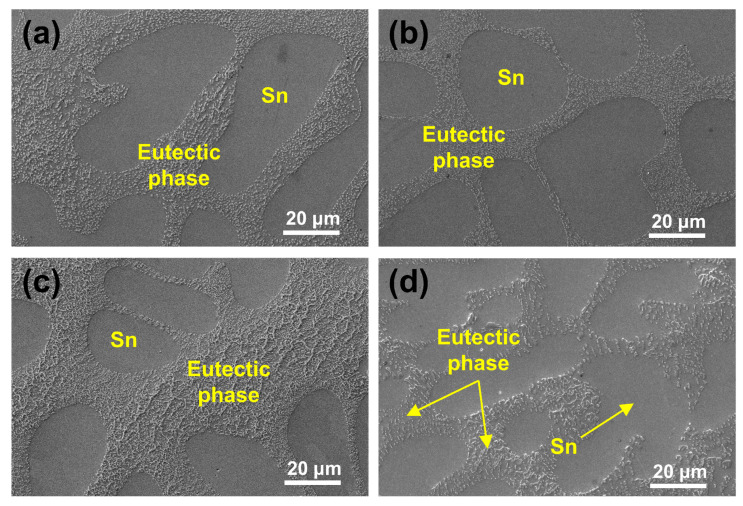
Morphologies within the grains of SAC305 under various conditions: (**a**) as-cast, (**b**) T75-D12, (**c**) T125-D12, and (**d**) T165-D12.

Figure 8 presents the microstructural morphologies within the grains of SAC0307 in its as-cast state and after various annealing treatments. In all the images, the phases indicated by the yellow arrows are the IMCs phases (including Cu_6_Sn_5_ and Ag_3_Sn) [58]. The microstructure of SAC0307 undergoes significant changes following annealing treatments, notably in the shape and size of the IMCs phase. To be pointed out, the size of IMCs in SAC0307 is too small to be accurately measured and thus will not be statistically analyzed. As the annealing temperature increases from 75 °C to 165 °C (Figure 9b–d), the IMCs phases progressively grow and coarsen, becoming more uniformly and distinctly distributed. Notably, the Cu_6_Sn_5_ IMC shows a significant increase in size, reaching a length of nearly 8 μm at 165 °C, as shown in Figure 8d. However, longer annealing durations result in minimal changes in the microstructure. For example, maintaining the annealing temperature at 125 °C and increasing the annealing duration from 6 h to 24 h (Figure 8e,c,f) shows negligible changes. This confirms that annealing duration has minimal influence on the microstructure evolution in SAC0307 alloy compared to annealing temperature. Combining the effect of the annealing duration on the size variation in Pb-rich phases in Sn63Pb37, it can be concluded that annealing duration is not a key factor influencing the microstructure evolution in solder alloy.

Figure 9 presents the microstructural morphologies within the grains of SAC305 in its as-cast state and after various annealing treatments. The microstructures of both as-cast and annealed SAC305 exhibit a typical eutectic phase with a reticular distribution. As confirmed in the literature, the eutectic phase contains two types of IMCs: Ag_3_Sn and Cu_6_Sn_5_ [59]. The images clearly show that annealing treatments have a significant impact on the size of the Sn matrix and the distribution of the eutectic phase in SAC305. As the annealing temperature increases from 75 °C to 165 °C, the size of the Sn matrix gradually decreases and the IMCs within the eutectic phase coarsen. This change is most pronounced at the annealing temperature of 165 °C, as seen in Figure 9d, where the eutectic phase IMCs exhibit further coarsening, resulting in a more sparse distribution of IMCs throughout the eutectic regions.

Based on the results discussed above, it is evident that annealing treatments significantly impact the microstructure of Sn63Pb37, SAC0307, and SAC305 solder alloys. These treatments primarily affect the shape, size, and distribution of the phases and grains. Increasing the annealing temperature and prolonging the annealing duration contribute to a more uniform distribution of microstructures and the coarsening of phases, such as the Pb-rich phase in Sn63Pb37 and the IMCs in SAC0307. These microstructural changes inevitably alter the mechanical properties of Sn63Pb37, SAC0307, and SAC305 solders, as further investigated through nano-indentation tests.

### 3.2. Impacts of Annealing Treatment on the Micro-Mechanical Properties

**Figure 10:**
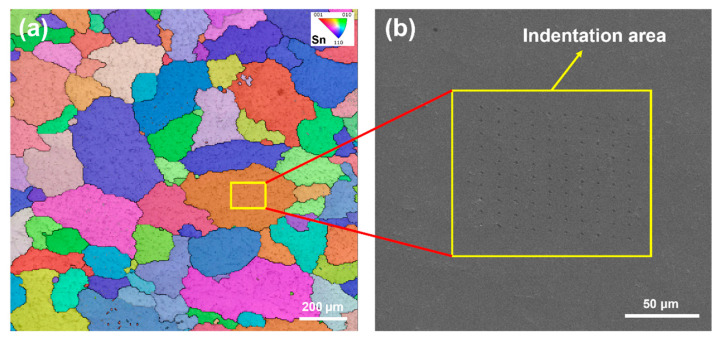
(**a**) Inverse pole figure and (**b**) indentation morphologies of a SAC0307 sample.

In this section, the nano-hardnesses and Young’s moduli of Sn63Pb37, SAC0307, and SAC305 solders were measured using nano-indentation tests. These tests were conducted on both as-cast samples and those subjected to various annealing conditions for each solder type. To enhance the reliability of the results and minimize potential errors, more than 15 effective indentations were performed on each sample. As previously verified, the average grain sizes of SAC0307, SAC305, and Sn63Pb37 alloys are 246.8 μm, 921.8 μm, and 149.9 μm, respectively, ensuring that current indentations were consistently located within the same grain. For instance, Figure 10 presents the IPF map of a SAC0307 sample, where a 10×10 indentation array had been performed with a spacing of 10 μm between indentations. This spacing ensures that the indentation area, highlighted within the yellow box, falls entirely within a single grain. Given the large grain size of the Sn phase in SAC305 and Sn63Pb37 samples, it also confirms that the indentation points also reside within the same grain. Therefore, the subsequent nano-indentation test results are independent of grain orientation.

**Figure 11:**
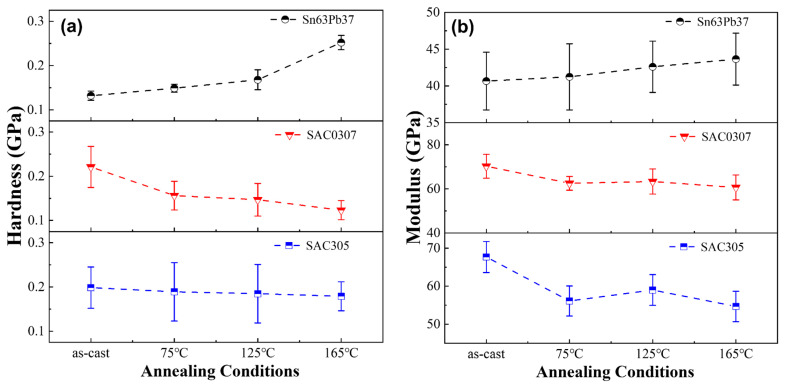
(**a**) Hardness and (**b**) modulus variations in three kinds of solder under different conditions (annealing duration: 12 h for all).

**Figure 12:**
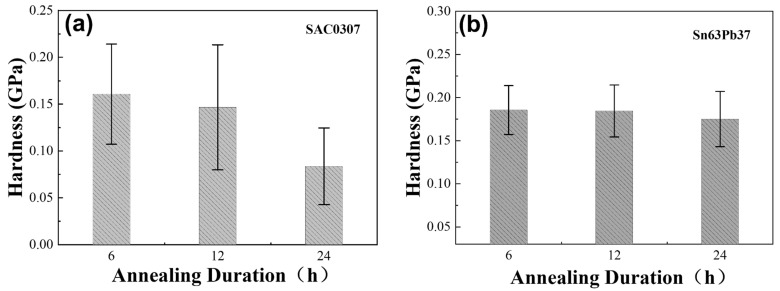
Hardnesses of (**a**) SAC0307 and (**b**) Sn63Pb37 under various annealing durations (fixed annealing temperature: 125 °C).

The average values of elastic moduli and hardnesses for Sn63Pb37, SAC0307, and SAC305 solders at different annealing temperatures are summarized. As depicted in Figure 11, initial measurements revealed differences in modulus and hardness among the three solder alloys in their as-cast state, with SAC0307 exhibiting the highest values and Sn63Pb37 the lowest. Notably, annealing treatments induce variations in the elastic modulus and hardness within each alloy. Specifically, for Sn63Pb37, both the average hardness and modulus increase with rising annealing temperature. In the reported studies [17,41], the tensile strength of eutectic Sn-Pb alloy was also found to increase significantly with the rise in annealing temperature. This observation highlights the consistency between the micromechanical and macro-mechanical properties of this solder alloy. In contrast, SAC0307 exhibits a gradual decrease in average hardness with increasing annealing temperature, along with a decrease in average modulus compared to its as-cast state. SAC305 demonstrates relatively stable hardness and modulus values across different annealing temperatures, although the modulus at all annealed states is lower than that of the as-cast state. These findings underscore the varying effect of annealing temperature on hardness and modulus among different solder alloys. Additionally, annealing duration also influences material properties. As seen from Figure 12, indentation tests on Sn63Pb37 and SAC0307 solder with varying annealing durations reveal that the average hardness of SAC0307 decreases progressively with longer annealing duration, with a notable reduction of 0.0629 GPa after 24 h compared to 12 h. Conversely, the average hardness of Sn63Pb37 alloy remained essentially unchanged with increased annealing duration.

Based on the results, it is concluded that the hardness of SAC0307 is sensitive to both annealing temperature and duration, whereas the hardness of Sn63Pb37 is highly sensitive to annealing temperature but not significantly affected by annealing duration. The differences in mechanical responses between SAC0307 and Sn63Pb37 are closely related to the effects of annealing treatments. As aforementioned, annealing temperature strongly impacts the microstructure within the grains of both alloys, resulting in significant variations in hardness. However, annealing duration has minimal effect on the evolution of the Pb-rich phase in Sn63Pb37 but greatly affects the evolution of IMC particles in SAC0307. Consequently, the hardness of Sn63Pb37 remains constant with different annealing durations, while in SAC0307, the significant coarsening of IMC particles with increasing annealing duration reduces the solder’s resistance to dislocations, leading to a decrease in hardness. As previously highlighted, IMC particles in SAC0307 inhibit the motion of dislocations [60].

As shown in Figure 12a, the hardness of SAC0307 solder exhibits a relatively large error. SAC0307 solder consists of the Sn matrix phase and IMCs phases. The indented point data obtained from statistical measurements include all phases present in the solder. The significant differences in properties between the Sn matrix phase and the IMCs phase contribute to the relatively large error in the measurement and statistical results. Therefore, it is necessary to further investigate the mechanical properties of each individual phase in the solder.

### 3.3. Mechanical Properties Variation in Individual Phase

During the annealing process, changes occur not only in the size and shape of individual solder phases but also in their mechanical properties. These individual phases, namely the Pb-rich phase and the Sn matrix in eutectic Pb-containing solder alloy, and the Sn phase, IMCs phase, and eutectic phase in Pb-free solder alloys, undergo significant alterations due to annealing treatment. Since these phases are small in size, macroscopic mechanical tests often fail to distinguish their independent mechanical properties. To overcome this limitation, we employed nano-indentation tests to successfully identify the distinct mechanical properties of each phase in both Pb-containing and Pb-free solder alloys.

**Figure 13:**
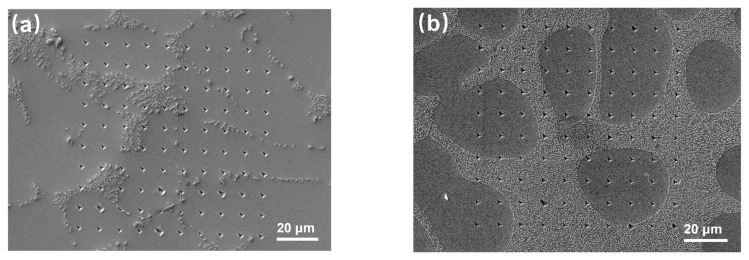
Images of indentation arrays on sample surface of (**a**) SAC0307 and (**b**) SAC305.

In Sn63Pb37, numerous Pb-rich phases and distinct Sn matrix regions are large enough (nearly 10 ${\mu m}^{2}$) to be clearly distinguished under an optical microscope, allowing for direct indentation testing on each individual phase. Since the plastic zone of a single indentation is no more than 1 ${\mu m}^{2},$ it is certain that the measured hardness for these individual phases is unaffected by their surroundings. To ensure the reliability of the tests, each phase in Sn63Pb37 underwent more than 20 indentations. For SAC0307 and SAC305, distinguishing the IMCs phase and the eutectic phase using the optical microscope on the testing platform proved challenging. Therefore, we employed an automated 10 × 10 indentation array on the sample surfaces, as illustrated in Figure 13. Subsequently, SEM was employed to identify the location of each indentation point to determine whether it was on the Sn matrix or the IMCs/eutectic phase. This approach allowed for the accurate screening of the mechanical properties of each individual phase. Consequently, the hardness for the individual phases in the three types of solder alloys were obtained and statistically averaged. To be pointed out, abnormal data from the 100 indentation points were excluded.

**Figure 14:**
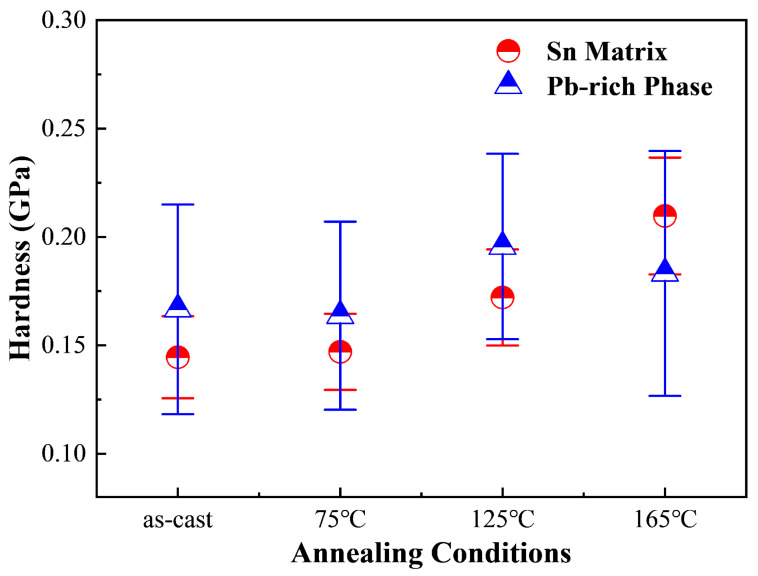
Hardness of Sn matrix and Pb-rich phase in Sn63Pb37 under various conditions (annealing duration: 12 h for all).

**Figure 15:**
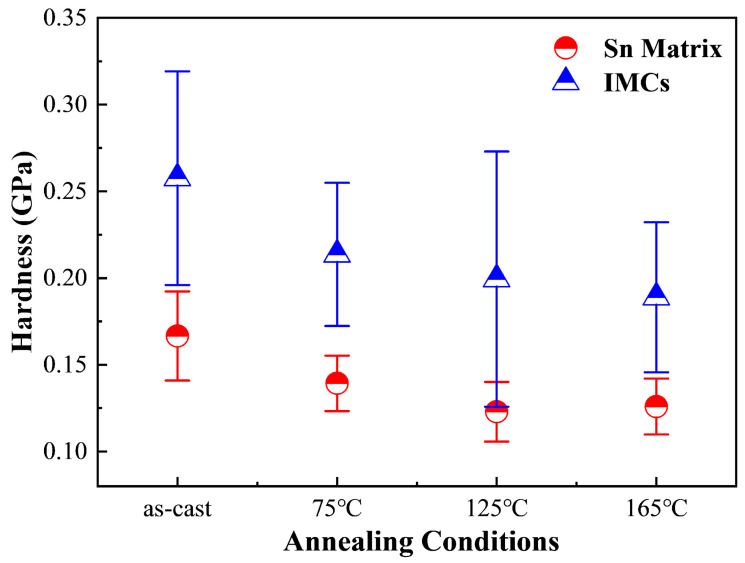
Hardness of Sn matrix and IMCs in SAC0307 under various conditions (annealing duration: 12 h for all).

As depicted in Figure 14, the average hardness of the Sn matrix in Sn63Pb37 alloy progressively increases with rising annealing temperature. In contrast, the hardness of the Pb-rich phase initially increases to 0.1956 GPa before declining to 0.1783 GPa at 165 °C. Therefore, the primary internal factor contributing to the overall hardness increase in Sn63Pb37 with higher annealing temperatures is the increased hardness of both the Sn and Pb-rich phases. Figure 15 illustrates the hardness of both the Sn matrix and IMCs phases in SAC0307 alloy at different annealing temperatures. Interestingly, both phases show a gradual decrease in average hardness as the annealing temperature increases, which contrasts with the behavior observed in Sn63Pb37. This difference further elucidates the pronounced decrease in hardness observed in SAC0307 solder at higher annealing temperatures, as depicted in Figure 11. As seen in Figure 14 and Figure 15, the hardness of the Sn matrix in SAC0307 and Sn63Pb37 alloys exhibits opposite behaviors with rising annealing temperatures. This phenomenon can be explained by the different evolutions of Sn matrix in them. The area fractions of the Pb-rich phase in Sn63Pb37 and IMCs phase in SAC0307 under different annealing conditions were obtained using an image processing tool.

**Figure 16:**
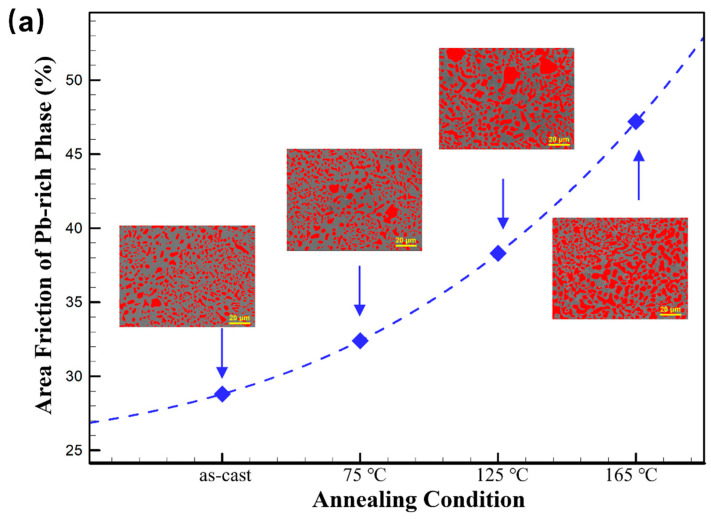

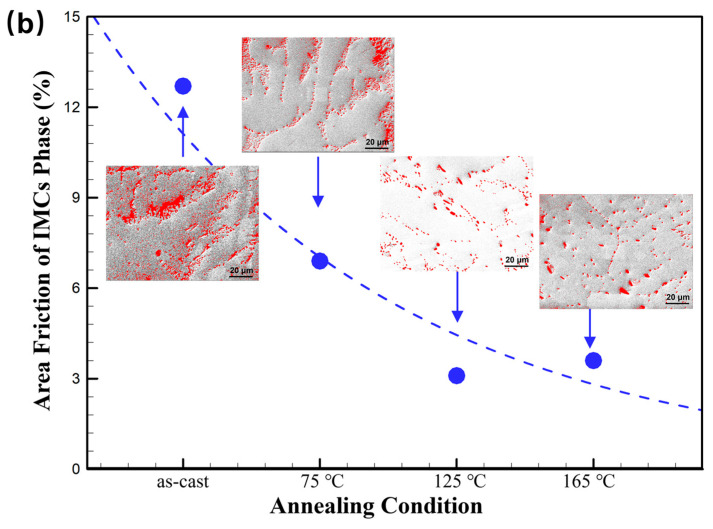
Area fraction of (**a**) Pb-rich phase in Sn63Pb37 and (**b**) IMCs phase in SAC0307 under various conditions (annealing duration: 12 h for all).

As shown in Figure 16a,b, the area fraction of the Pb-rich phase in Sn63Pb37 increases significantly with higher annealing temperatures, while the area fraction of the IMCs in SAC0307 gradually decreases. Consequently, the area of the Sn matrix phase in Sn63Pb37 decreases with increasing annealing temperatures. Due to the reduced free volume at higher annealing temperature, dislocations produced by the indentation are more easily confined by the Sn/Pb phase boundaries, resulting in the increasing hardness of the Sn matrix phase in Sn63Pb37. In contrast, in SAC0307, the area fraction of the Sn matrix phase increases with higher annealing temperatures, resulting in more free volume for the dislocation movement. Thus, the hardness of the Sn matrix phase in it decreases with increasing annealing temperatures.

**Figure 17:**
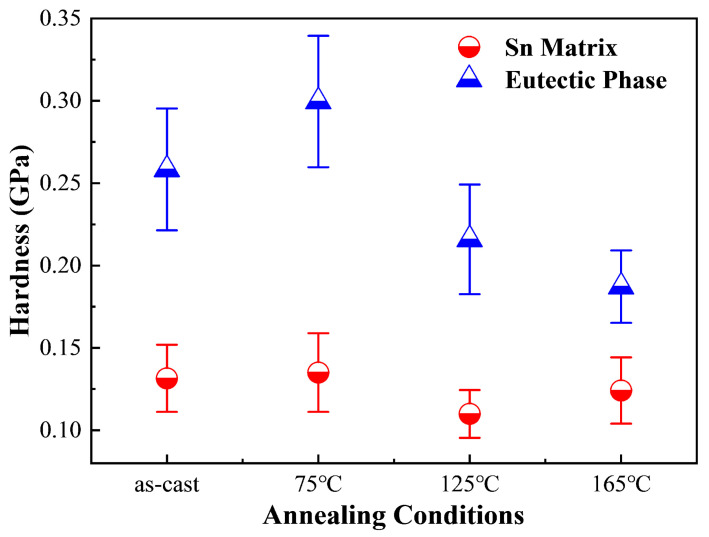
Hardness of Sn matrix and eutectic phase in SAC305 under various conditions (annealing duration: 12 h for all).

**Figure 18:**
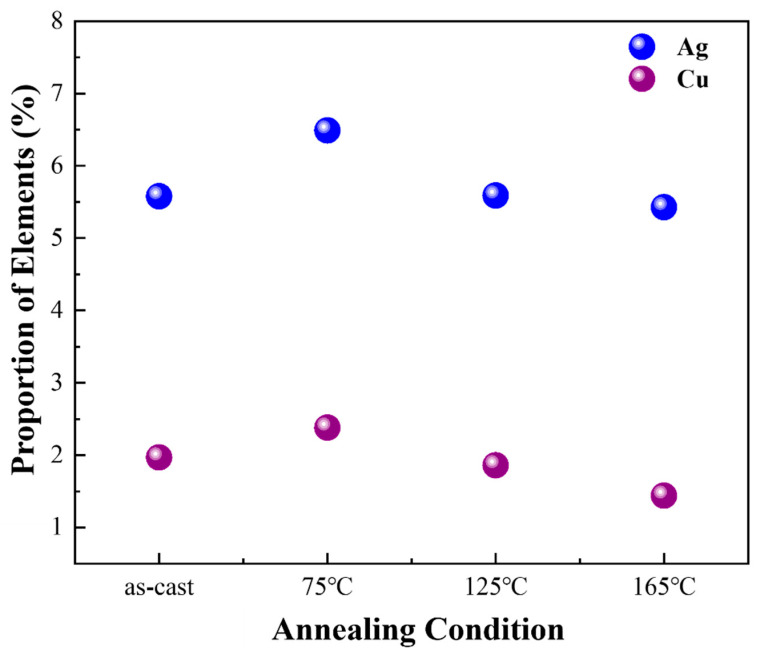
Proportion of Ag and Cu in the eutectic phase in SAC305 under various conditions (annealing duration: 12 h for all).

The average hardness of the Sn matrix and eutectic phase in SAC305 alloy at various annealing temperatures is shown in Figure 17. It can be observed that the hardness of the Sn matrix remains relatively stable across different annealing temperatures. This consistency can be attributed to the substantial size of the Sn matrix in SAC305, which allows for multiple indentations, as illustrated in Figure 13b. Consequently, the plastic deformation caused by each indentation remains unaffected by the surroundings, ensuring the unaltered hardness of the Sn matrix in SAC305. However, the hardness of the eutectic phase shows a slight increase but then declines with increasing temperature. To elucidate this behavior, EDS analysis was performed on the elemental composition of the eutectic phase in SAC305 under various conditions. Notably, the variations in the proportions of Ag and Cu, which correspond to the content of IMCs (Ag_3_Sn and Cu_6_Sn_5_) under different conditions, align with the observed changes in hardness of the eutectic phase, as illustrated in Figure 17 and Figure 18. Ag and Cu elements are indicative of the content of IMCs, which contribute to hardness. Therefore, a decrease in their proportions with increasing annealing temperature results in a reduced hardness of the eutectic phase. This also explains the observed decrease in the hardness of IMCs in SAC0307.

**Figure 19:**
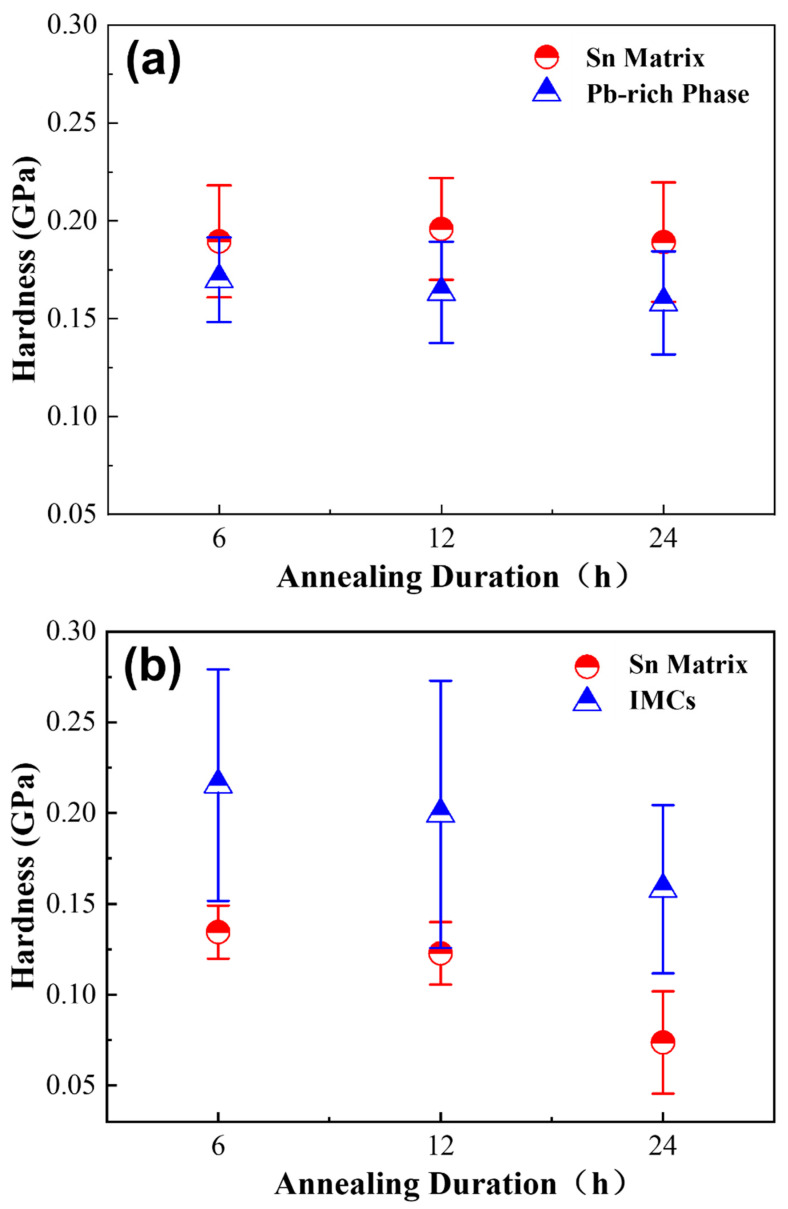
Hardness of individual phases in (**a**) Sn63Pb37 and (**b**) SAC0307 at annealing temperature of 125 °C but different durations.

Figure 19a shows that the hardness of both the Sn and Pb-rich phases in Sn63Pb37 remains relatively stable with increasing annealing duration, with the Sn phase exhibiting slightly higher hardness than the Pb-rich phase. This stability partially accounts for the minimal change in the overall hardness of Sn63Pb37 alloy with varying annealing durations. In contrast, Figure 19b illustrates a notable decrease in the average hardness of both the Sn matrix and IMCs phases in SAC0307 solder with prolonged annealing duration. According to Table 1, the proportion of IMCs phase observed during indentation decreases from 32.3% at 6 h to 11.8% at 24 h. Given that the IMCs phase has relatively high hardness, its reduced proportion contributes to the observed decrease in average hardness, thereby explaining the significant reduction in the hardness of SAC0307 solder with extended annealing durations.

## 4. Conclusions

This study investigated both Pb-containing and Pb-free solder alloys subjected to various annealing temperatures and durations. The microstructures of these alloys were characterized using SEM and EBSD, while nano-indentation experiments were conducted to evaluate their mechanical properties. The study elucidated the effects of annealing treatments on mechanical properties through changes in microstructure. The main conclusions are as follows:

(1) Annealing significantly altered the microstructures of Sn63Pb37, SAC0307, and SAC305 solders. For SAC0307 and SAC305, grain size increased with higher annealing temperatures, while Sn63Pb37 exhibited less pronounced grain size changes. Both elevated temperature and extended duration promoted coarsening of the Pb-rich phase in Pb-containing solders and IMCs in Pb-free solders.
(2) The hardness of different solder alloys responded differently to annealing. The hardness of Sn63Pb37 increased with rising annealing temperature and was insensitive to annealing duration. In contrast, the hardness of SAC0307 decreased with increasing temperature and extended duration, while that of SAC305 remained relatively stable.
(3) The distinct hardness behaviors were attributed to the differing evolutions of the Sn matrix. In Sn63Pb37, the expansion of the Pb-rich phase limited dislocation motion, increasing the hardness of the Sn matrix. Conversely, in SAC0307, the enlarged Sn matrix provided more free volume for dislocation motion, reducing hardness.

## Figures and Tables

**Table 1:** Proportion of indentations on IMCs phases in different SAC0307 sample.

Type of Sample	Proportion of IMCs (%)
125 °C—6 h	32.3
125 °C—12 h	25.6
125 °C—24 h	11.8

## Data Availability

The original contributions presented in this study are included in the article. Further inquiries can be directed to the corresponding author.
